# A Retrospective Analysis of Standardized Gradient Calculations for Evaluating Patient–Prosthesis Mismatch Following Mechanical Aortic Valve Replacement

**DOI:** 10.3390/diagnostics15050567

**Published:** 2025-02-26

**Authors:** Muhammet Fethi Sağlam, Emrah Uguz, Kemal Eşref Erdogan, Hüseyin Ünsal Erçelik, Murat Yücel, Altay Alili, Nur Gizem Elipek, Okay Güven Karaca, Erol Şener

**Affiliations:** 1 Department of Cardiovascular Surgery, Faculty of Medicine, Ankara Yıldırım Beyazıt University, 06000 Ankara, Türkiye; emrahuguz@gmail.com (E.U.); kemal_esref@hotmail.com (K.E.E.); alilialtay97@gmail.com (A.A.); gizelipek@gmail.com (N.G.E.); drerolsener@gmail.com (E.Ş.); 2 Ankara Bilkent City Hospital, 06800 Ankara, Türkiye; unsalercelik@gmail.com (H.Ü.E.); dr_yucelmurat@hotmail.com (M.Y.); 3 İzmir City Hospital, 35510 İzmir, Türkiye; drguven@gmail.com

**Keywords:** aortic stenosis, aortic valve replacement, transvalvular gradient, patient–prosthesis mismatch, standardized gradient

## Abstract

**Background:** Aortic stenosis (AS) is one of the most common valvular heart diseases, particularly in the elderly, with a prevalence of approximately 3% in individuals over 75 years of age. Aortic valve replacement (AVR) remains the standard treatment, yet postoperative hemodynamic assessment is often complicated by variations in prosthetic valve size, left ventricular ejection fraction (LVEF), effective orifice area (EOA), and body surface area (BSA). These factors significantly influence prosthetic valve function and contribute to patient–prosthesis mismatch (PPM), which has been associated with worse clinical outcomes. Traditional transvalvular gradient measurements often fail to account for these patient-specific variables. This study introduces a novel approach to standardized gradient calculations, aiming to enhance the accuracy and comparability of prosthetic valve assessments. **Methods**: A retrospective analysis was conducted on 115 patients who underwent mechanical AVR at a single center. Patients were categorized into three groups based on the prosthetic valve type: St. Jude Medical (SJM) HP (*n* = 31); SJM Regent (*n* = 54); and those who underwent aortic root enlargement (ARE) (*n* = 30). Preoperative and postoperative transthoracic echocardiography (TTE) was performed to measure conventional and standardized transvalvular gradients. Four novel standardized gradient calculations were developed to adjust for individual hemodynamic differences, improving the accuracy of prosthetic valve function assessment. **Results**: Standardized gradient calculations demonstrated significant differences between prosthesis types. Postoperative standardized gradients were significantly higher in the SJM HP group compared to the SJM Regent and aortic-root-enlargement groups (*p* < 0.001, *p* < 0.05). The lowest standardized gradients were observed in patients who received the SJM Regent prostheses (*p* < 0.05). Although conventional measurements showed no significant differences, standardized calculations revealed that patients with 19 mm prostheses exhibited significantly higher transvalvular gradients than those with 21 mm prostheses (*p* < 0.05), emphasizing the clinical importance of prosthesis size in postoperative hemodynamics. **Conclusions**: Standardized gradient calculations provide a more objective, reliable, and patient-specific assessment of prosthetic valve function by minimizing interpatient variability. This approach improves the detection of patient–prosthesis mismatch and optimizes postoperative hemodynamic evaluation, potentially leading to better prosthesis selection and surgical decision-making. However, further validation is required in larger cohorts before these methods can be widely adopted into clinical practice. Future studies should assess their impact on long-term clinical outcomes, including left ventricular remodeling and patient survival.

## 1. Introduction

Aortic stenosis (AS) is one of the most prevalent valvular heart diseases, particularly in the elderly population, affecting approximately 3% of individuals over the age of 75 [1]. If left untreated, severe AS is associated with a high mortality rate, with an estimated 50% survival at two years once symptoms appear [2]. Aortic valve replacement (AVR) remains the standard treatment, either via surgical AVR (SAVR) or transcatheter AVR (TAVR), both of which have been shown to significantly improve survival and quality of life [3]. Despite advances in prosthetic valve technology, postoperative hemodynamic assessment remains a major challenge in clinical practice as conventional methods may not fully account for interpatient variability [4].

The functional success of aortic valve prostheses is typically assessed using maximum and mean transvalvular gradient measurements obtained through transthoracic echocardiography (TTE) [5]. However, these parameters are strongly influenced by prosthetic valve size, left ventricular ejection fraction (LVEF), body surface area (BSA), and prosthesis cover diameter, leading to significant variability in hemodynamic performance interpretation [6,7]. This variability is particularly problematic in patients with low LVEF (<50%), where it becomes difficult to distinguish between true stenosis and patient–prosthesis mismatch (PPM) [8]. Therefore, reliance on absolute transvalvular gradient values may not provide sufficient clinical insight.

PPM is a critical issue in prosthetic valve function assessment, occurring when the effective orifice area (EOA) of the prosthetic valve is disproportionately small relative to the patient’s BSA, resulting in elevated postoperative transvalvular gradients [9]. PPM is particularly prevalent in patients receiving smaller prosthetic valves as these may not provide sufficient hemodynamic performance [10]. Current clinical practice primarily assesses PPM by dividing the prosthetic valve’s EOA by the patient’s BSA; however, this method fails to incorporate other critical hemodynamic variables, such as LVEF and prosthetic cover diameter, which significantly influence postoperative transvalvular gradients and overall left ventricular function [11,12].

Several studies have explored the impact of prosthetic valve selection and implantation techniques on hemodynamic outcomes. For instance, prior research has investigated left ventricular systolic function improvement after TAVR in patients with low-flow, low-gradient, severe AS, emphasizing the importance of optimizing hemodynamics in AS management [13,14]. Additionally, studies have demonstrated the potential benefits of alternative valve replacement techniques, such as sutureless aortic valve replacement, in reducing transvalvular gradients [15,16]. However, these studies have primarily focused on procedural outcomes and short-term hemodynamics rather than addressing the need for a standardized method to evaluate transvalvular gradients across different patient profiles [17,18].

To address these limitations, we developed a novel standardized gradient calculation method to minimize the influence of interpatient hemodynamic variations. These standardized calculations provide a more objective and comparable approach for evaluating prosthetic valve function by ensuring that BSA, LVEF, prosthetic EOA, valve size, and aortic root diameter are appropriately considered in gradient measurements.

This study aimed to calculate preoperative and postoperative standardized transvalvular gradients in patients undergoing mechanical AVR and compare these standardized values with conventional transvalvular gradient measurements. Furthermore, we sought to evaluate the effectiveness of this novel calculation method across different prosthetic valve types, specifically St. Jude Medical (SJM) HP and SJM Regent, as well as in patients who underwent aortic root enlargement (ARE) procedures. By addressing these gaps, our study provides a more accurate and clinically useful method for assessing prosthetic valve function and optimizing surgical decision-making in patients with AS. The integration of standardized gradient calculations into routine clinical practice may improve prosthesis selection, patient outcomes, and the early identification of PPM, potentially reducing long-term cardiovascular complications.

## 2. Materials and Methods

### 2.1. Study Design and Ethical Approval

This study was conducted at the Cardiovascular Surgery Clinic of Ankara Bilkent City Hospital. Ethical approval was obtained from the 1st Clinical Research Ethics Committee of Ankara Bilkent City Hospital on 17 July 2024, with decision number TABED 1-24-382. This study was reviewed and unanimously approved in terms of ethical considerations.

The research was carried out in accordance with the principles of the Declaration of Helsinki, ensuring the ethical treatment of all participants. The confidentiality of patient data was fully protected throughout this study, and informed consent was obtained from all participants prior to inclusion in the research.

### 2.2. Patient Selection and Study Population

A total of 115 patients who underwent mechanical aortic valve replacement for severe aortic stenosis between 2019 and 2024 were included in this study. Patients were divided into three groups according to the type of prosthetic valve implanted and surgical techniques performed. The first group consisted of 31 patients implanted with the SJM HP mechanical aortic valve. The second group consisted of 54 patients implanted with the SJM Regent mechanical aortic valve. The third group consisted of 30 patients who underwent valve replacement by aortic root enlargement procedure.

All surgical procedures were performed by experienced surgeons at the Cardiovascular Surgery Clinic of Ankara Bilkent City Hospital.

### 2.3. Inclusion Criteria

The patients included in this study were selected according to certain criteria. First, patients had to be diagnosed with severe aortic stenosis (AS). The diagnosis was confirmed by echocardiographic evaluations. Patients with an aortic valve area (AVA) ≤ 1.0 cm^2^, mean transvalvular gradient ≥ 40 mmHg, or peak aortic jet velocity ≥ 4.0 m/s were included. The primary surgical procedure was mechanical AVR. Some patients additionally underwent an aortic root dilatation procedure. Transthoracic echocardiography (TTE) was used for preoperative and postoperative evaluation. Echocardiographic measurements were performed in accordance with international guidelines. In addition, the complete clinical records were available and informed consent was obtained for participation in this study.

### 2.4. Exclusion Criteria

Patients with certain clinical and surgical characteristics were excluded to ensure the accuracy and reliability of the standardized gradient calculations. Patients with previous aortic valve surgery were excluded as prior interventions could influence hemodynamic outcomes. Likewise, patients requiring additional surgical procedures for aortic aneurysm or severe aortic insufficiency were excluded to minimize confounding factors. Patients undergoing concomitant valve surgery for mitral or tricuspid valve disease were excluded due to the potential impact on transvalvular gradients and left ventricular hemodynamics. Similarly, patients with a history of infective endocarditis or evidence of active infection during surgery were excluded to eliminate potential inflammatory effects on postoperative outcomes. To control for the influence of postoperative complications on standardized gradient measurements, patients who developed severe postoperative complications that could affect hemodynamics were excluded. This included patients who experienced perioperative mortality, required urgent surgical revision due to hemodynamic instability, or developed mechanical valve dysfunction in the early postoperative period. Additionally, patients with prolonged low cardiac output syndrome, significant paravalvular leakage, or new-onset severe arrhythmias (such as atrial fibrillation with rapid ventricular response) were excluded as these conditions could introduce variability in the echocardiographic gradient assessments.

Furthermore, patients with incomplete or inadequate clinical and echocardiographic data were excluded to ensure methodological consistency. Missing data were handled by excluding cases where key parameters required for standardized gradient calculations—such as left ventricular ejection fraction (LVEF), effective orifice area (EOA), or body surface area (BSA)—were unavailable or unreliable.

#### 2.4.1. Data Collection Process

In this retrospective study, demographic, clinical, and echocardiographic data of the patients were obtained from the hospital’s patient tracking system and surgical records. The data collection process was carried out in two phases.

#### 2.4.2. Collection of Demographic and Clinical Data

The age, gender, height, weight, and body mass index (BMI) of each patient were recorded from the hospital database. Body surface area (BSA) was also calculated for all patients.

The presence of comorbidities such as hypertension (HT), diabetes mellitus (DM), chronic kidney disease (CKD), and chronic obstructive pulmonary disease (COPD) was identified based on the patients’ medical records.

Surgical details, including the type of implanted mechanical prosthetic valve and additional procedures performed, were collected. Since all prosthetic valves used in this study were mechanical (metallic) valves from the same manufacturer—St. Jude Medical^®^ Mechanical Heart Valves (SJM; St. Jude Medical Inc., Minneapolis, MN, USA)—patients were classified based on the specific model of the implanted valve. Patients were grouped separately as those receiving SJM HP and SJM Regent prosthetic valves. Additionally, patients who underwent aortic root enlargement along with valve replacement were analyzed as a distinct group.

#### 2.4.3. Echocardiographic Measurements

All patients underwent preoperative and postoperative transthoracic echocardiography (TTE). The measurements were performed by experienced cardiologists in accordance with the guidelines of the American Society of Echocardiography (ASE).

Ejection fraction (EF) was assessed using Simpson’s method, which is the recommended technique for evaluating left ventricular systolic function. The aortic root diameter was measured from parasternal long-axis views. Left ventricular septal wall thickness and free wall thickness were recorded during diastole. Transvalvular gradients were measured, and the maximal transvalvular gradient (MaxG) and mean transvalvular gradient (MeanG) were calculated using the continuity equation. Gradient measurements were obtained using the continuous-wave Doppler (CW Doppler) technique, ensuring consistency across all assessments.

#### 2.4.4. Inter-Observer and Intra-Observer Variability

To ensure measurement consistency and minimize variability, all echocardiographic assessments were conducted by two independent cardiologists, each with at least five years of experience in echocardiographic evaluation. Intra-observer variability was assessed by having the same cardiologist reanalyze 20 randomly selected transthoracic echocardiography (TTE) scans at least two weeks apart, ensuring they were blinded to the initial results. This approach aimed to evaluate the repeatability of measurements taken by the same observer. Inter-observer variability was evaluated by comparing gradient measurements from two independent cardiologists on another set of 20 randomly selected TTE scans, also in a blinded manner, to determine the consistency between different observers. Intraclass correlation coefficient (ICC) analysis was performed to assess both intra- and inter-observer reliability. The ICC values for the maximum gradient (MaxG) and mean gradient (MeanG) were 0.89 and 0.91, respectively, indicating excellent agreement. Any discrepancies exceeding a 10% variation were resolved through a consensus review with a third senior cardiologist. These measures ensured that observer-related variability had minimal impact on the transvalvular gradient assessments, thereby enhancing the reliability of the study findings.

#### 2.4.5. Standardized Gradient Calculations

In this study, conventional transvalvular gradient values were standardized to account for individual hemodynamic variations, such as ejection fraction (EF), body surface area (BSA), prosthetic valve size, and effective orifice area (EOA). The goal of this standardization was to provide a more objective and patient-specific assessment of prosthetic valve function by minimizing the impact of interpatient variability. Standardized gradient calculations were performed separately for preoperative and postoperative measurements using four distinct formulas.

### 2.5. Rationale for the Selected Standardization Methods

Transvalvular gradient measurements are highly dependent on patient-specific anatomical and physiological characteristics. Previous studies have attempted to index gradients based on EOA or BSA alone but these approaches often fail to account for the combined influence of LVEF, prosthetic valve size, and aortic dimensions. The four standardization formulas in this study were chosen to adjust the transvalvular gradients for key hemodynamic parameters, ensuring comparability across the different cardiac outputs and body sizes; this helps to distinguish preoperative and postoperative conditions by considering prosthetic valve function and incorporates prosthetic valve diameter, recognizing its impact on hemodynamic performance.

### 2.6. Body Surface Area (BSA) Consideration

BSA was incorporated into all gradient calculations to normalize for patient size and cardiac output differences. Since BSA varies with height and weight, using it as a scaling factor allowed for a more accurate comparison between patients with different body morphologies. This adjustment was particularly important in patients with a small annulus, where transvalvular gradients tend to be overestimated.

### 2.7. Validation and Internal Consistency

To ensure the reliability of these calculations, several validation steps were undertaken. First, a comparative analysis was performed by comparing standardized values with conventional gradients to determine whether normalization effectively reduced interpatient variability. Additionally, subgroup comparisons were conducted to assess the effectiveness of the formulas across different prosthetic valve types (SJM HP vs. SJM Regent) and sizes (19 mm vs. 21 mm). Finally, statistical verification was carried out using intraclass correlation coefficients (ICC) to evaluate the reproducibility and internal consistency. These validation steps confirmed that the proposed calculations provided a more accurate and unbiased assessment of prosthetic valve function.

### 2.8. Preoperative Standardized Maximum Gradient

The preoperative standardized maximum transvalvular gradient was calculated to adjust the conventional maximum gradient values based on body surface area, aortic root diameter, effective orifice area, and ejection fraction. This standardization allowed for a more accurate comparison of transvalvular gradients across different patients by minimizing the effect of individual anatomical and hemodynamic differences.

$$Std\text{-}MaxG\;Preop=\frac{MaxG\;Preop\times D(Ao)}{AVA\times LVEF\times BSA}$$

where Std-MaxG Preop is the preoperative standardized maximum gradient; MaxG Preop is the preoperative maximum transvalvular gradient; D(Ao) is the aortic root diameter; BSA is the body surface area; AVA is the aortic valve area; and LVEF is the left ventricular ejection fraction.

#### Preoperative Standardized Mean Gradient

The preoperative standardized mean transvalvular gradient was determined using a similar approach, incorporating patient-specific factors to normalize mean gradient values. By applying this calculation, the variability caused by differences in prosthetic valve size and cardiac output was reduced, leading to a more consistent interpretation of preoperative hemodynamics.

$$Std\text{-}MeanG\;Preop=\frac{MeanG\;Preop\times D(Ao)}{AVA\times LVEF\times BSA}$$

where Std-MeanG Preop is the preoperative standardized mean gradient; MeanG Preop is the preoperative mean transvalvular gradient; D(Ao) is the aortic root diameter; BSA is the body surface area; AVA is the aortic valve area; and LVEF is the left ventricular ejection fraction.

### 2.9. Postoperative Standardized Maximum Gradient

The postoperative standardized maximum transvalvular gradient was computed to evaluate the performance of the implanted prosthetic valve while accounting for changes in ejection fraction, effective orifice area, and body surface area. This calculation provided a more reliable assessment of postoperative hemodynamics by mitigating the confounding effects of individual physiological differences.

$$Std\text{-}MaxG\;Postop=\frac{MaxG\;Postop\times D(Pr)}{EOA\times LVEF\times BSA}$$

where Std-MaxG Postop is the postoperative standardized maximum gradient; MaxG Postop is the postoperative maximum transvalvular gradient; D(Pr) is the prosthetic valve diameter; BSA is the body surface area; EOA is the effective orifice area; and LVEF is the left ventricular ejection fraction.

#### Postoperative Standardized Mean Gradient

The postoperative standardized mean transvalvular gradient was used to assess the overall transvalvular pressure load after valve implantation. By incorporating standardized adjustments, this measurement allowed for a more precise comparison of postoperative mean gradient values among different patient groups, regardless of variations in cardiac output and prosthetic valve size.

$$Std\text{-}MeanG\;Postop=\frac{MeanG\;Postop\times D(Pr)}{EOA\times LVEF\times BSA}$$

where Std-MeanG Postop is the postoperative standardized mean gradient; MeanG Postop is the postoperative mean transvalvular gradient; D(Pr) is the prosthetic valve diameter; BSA is the body surface area; EOA is the effective orifice area; and LVEF is the left ventricular ejection fraction.

### 2.10. Statistical Analysis

Statistical analyses were performed to evaluate the differences among patient groups and within individual groups before and after surgery. Descriptive statistics for continuous variables were presented as mean ± standard deviation, median, and minimum–maximum values, while categorical variables were expressed as frequencies and percentages. The Kolmogorov–Smirnov test was used to assess the normality of data distribution. For the comparison of continuous variables among patients receiving SJM HP, SJM Regent, and those undergoing aortic root enlargement, one-way Analysis of Variance (ANOVA) was used for normally distributed data, while the Kruskal–Wallis variance analysis was applied for non-normally distributed data. Wilcoxon’s test was used to compare preoperative and postoperative measurements within the same group. The Mann–Whitney U test was applied for the comparison of continuous variables between two independent groups. For categorical variables, the Chi-Square test and Fisher’s exact test were used where applicable to compare distributions among groups. A priori power analysis was conducted using G*Power (version 3.1.9.7) software to determine the appropriate sample size. The analysis was based on effect size estimations derived from previous studies [19,20] on hemodynamic outcomes following aortic valve replacement. With an alpha level of 0.05 and a power (1 − β) of 0.80, a minimum sample size of 24 patients per group was determined to be sufficient to detect clinically meaningful differences. All statistical analyses were conducted using IBM SPSS for Windows 20.0 (SPSS Inc., Chicago, IL, USA), and a *p*-value of <0.05 was considered statistically significant.

### 2.11. Missing Data Handling

Missing data were handled systematically to minimize bias and maintain analytical robustness. Cases with missing key variables required for standardized gradient calculations, such as left ventricular ejection fraction (LVEF), effective orifice area (EOA), and body surface area (BSA), were excluded from the final analysis. For categorical variables, missing data were assessed for randomness using the Little’s Missing Completely at Random (MCAR) test. Since missing data were minimal (≤5% of the total dataset), no imputation techniques were required. However, in cases where individual echocardiographic measurements were unavailable, listwise deletion was applied to maintain internal consistency.

To evaluate the potential impact of missing data, sensitivity analyses were performed comparing results from complete cases to those with minor missing values. These analyses confirmed that the missing data did not significantly affect overall findings.

## 3. Results

A total of 115 patients were included in this study. The mean age of the patients was 55.25 ± 13.66 years, with the youngest patient being 18 years old and the oldest 76 years old. The mean body mass index (BMI) was 27.58 ± 4.77 kg/m^2^, and the mean body surface area (BSA) was 1.79 ± 0.15 m^2^. Of the patients, 54.8% were male and 45.2% were female. The morbidity rate was 4.3%, while 95.7% of the patients had no additional comorbidities. Hypertension (HT) was present in 66.1% of the patients; diabetes mellitus (DM) in 26.1%; chronic kidney disease (CKD) in 3.5%; and chronic obstructive pulmonary disease (COPD) in 13.9%. According to the New York Heart Association (NYHA) classification, 30.7% of the patients were in class 1, 60.5% in class 2, and 8.8% in class 3. Regarding prosthetic valve sizes, 21.7% of the patients received a 19 mm prosthetic valve, while 78.3% received a 21 mm valve. Based on the type of prosthetic valve used, 47% of the patients received an SJM Regent valve, 27% received an SJM HP valve, and 26.1% underwent aortic root enlargement (ARE) along with valve replacement. Preoperative echocardiographic measurements showed that the mean aortic root diameter was 2.27 ± 1.67 cm, with a median value of 2.1 cm (range: 1.8–2.0 cm). The mean aortic valve area (AVA) was 0.74 ± 0.15 cm^2^, while the mean effective orifice area of the prosthetic valve was 1.71 ± 0.29 cm^2^.

Table 1 presents the demographic, clinical, and preoperative echocardiographic characteristics of patients undergoing aortic valve replacement with SJM HP, SJM Regent prosthetic valves, or aortic root enlargement.

There were no significant differences among the three groups in terms of age, height, weight, body mass index, body surface area, preoperative aortic root diameter, and preoperative aortic valve area (*p* > 0.05).

A significant difference was observed in the prosthetic effective orifice area among the groups (*p* < 0.05). Patients receiving SJM Regent prostheses had a significantly larger prosthetic effective orifice area compared to both the SJM HP group (*p* < 0.001) and the aortic root enlargement group (*p* < 0.05). Additionally, the prosthetic effective orifice area was significantly greater in the aortic root enlargement group compared to the SJM HP group (*p* < 0.001).

A significant difference was found in gender distribution among the groups (*p* < 0.05). The proportion of female patients was higher in the aortic root enlargement group compared to both the SJM HP (*p* = 0.003) and SJM Regent (*p* = 0.034) groups.

No significant differences were detected among the three groups regarding comorbidities, including hypertension, diabetes mellitus, chronic kidney disease, and chronic obstructive pulmonary disease (*p* > 0.05).

Similarly, there was no statistically significant difference in prosthesis sizes among the groups (*p* > 0.05).

Based on Table 2, no significant difference was observed between preoperative and postoperative left ventricular ejection fraction (LVEF) values in patients who underwent SJM HP and aortic root enlargement procedures (*p* > 0.05). However, a significant decrease in postoperative LVEF compared to preoperative values was detected in the SJM Regent group (*p* < 0.05). Among the three groups, postoperative LVEF values were similar (*p* > 0.05), and the change in LVEF (Δ LVEF) between preoperative and postoperative periods did not significantly differ (*p* > 0.05).

Table 2 also indicates a significant reduction in maximal gradient values postoperatively in all three groups (*p* < 0.001). Patients who underwent SJM HP, SJM Regent implantation, and aortic root enlargement all exhibited lower postoperative maximal gradient values compared to preoperative levels. Despite these reductions, no significant difference was found in postoperative maximal gradient values among the three groups (*p* > 0.05). Additionally, the change in maximal gradient values (Δ maximal gradient) between preoperative and postoperative periods remained similar among the groups (*p* > 0.05), suggesting a comparable level of gradient reduction across all patients.

Preoperative mean gradient values were significantly higher than postoperative mean gradient values in all groups (*p* < 0.001), confirming that each surgical approach effectively lowered the mean transvalvular gradient. However, no significant differences were found in postoperative mean gradient values among the three groups (*p* > 0.05). Similarly, the change in mean gradient values (Δ mean gradient) between preoperative and postoperative periods was comparable among the groups (*p* > 0.05).

Findings from Table 2 demonstrate that septal wall thickness significantly decreased postoperatively in all three groups (*p* < 0.001), indicating structural changes in ventricular morphology. However, postoperative septal wall thickness values were similar among the three groups, with no significant differences detected (*p* > 0.05). Additionally, the change in septal wall thickness (Δ septal thickness) between preoperative and postoperative measurements did not significantly differ among the groups (*p* > 0.05), indicating a consistent level of postoperative remodeling.

Similarly, ventricular free wall thickness showed a significant reduction in all three groups (*p* < 0.001 for SJM HP and SJM Regent; *p* < 0.05 for aortic root enlargement). However, no significant differences were found in postoperative ventricular free wall thickness values among the three groups (*p* > 0.05). Moreover, the change in ventricular free wall thickness (Δ posterior wall thickness) was not significantly different among the groups (*p* > 0.05), suggesting a uniform degree of myocardial remodeling following surgery.

Standardized maximal gradient values also showed a significant postoperative reduction across all three groups (*p* < 0.001). However, postoperative standardized maximal gradient values were significantly higher in the SJM HP group compared to both the SJM Regent and aortic root enlargement groups (*p* < 0.001 and *p* < 0.01, respectively). Despite this, the change in standardized maximal gradient values (Δ standardized maximal gradient) did not significantly differ among the groups (*p* > 0.05), indicating that all patients experienced a similar level of reduction.

Similarly, standardized mean gradient values significantly decreased postoperatively in all groups (*p* < 0.001). Nevertheless, postoperative standardized mean gradient values were significantly higher in the SJM HP group compared to both the SJM Regent and aortic root enlargement groups (*p* < 0.001 and *p* < 0.05, respectively). Despite these differences, the change in standardized mean gradient values (Δ standardized mean gradient) was comparable among the groups (*p* > 0.05), suggesting a similar degree of gradient reduction across all three surgical techniques.

Based on Table 3, no significant differences were observed in postoperative left ventricular ejection fraction, postoperative maximal gradient, postoperative mean gradient, postoperative septal thickness, and postoperative ventricular free wall thickness between patients who received SJM Regent prostheses of 19 mm and those who received 21 mm prostheses (*p* > 0.05).

However, a significant difference was found in postoperative standardized maximal gradient values between the two prosthesis sizes (*p* < 0.05). Patients with 19 mm prosthetic valves exhibited significantly higher postoperative standardized maximal gradient values compared to those with 21 mm prosthetic valves.

Similarly, a significant difference was detected in postoperative standardized mean gradient values between the two prosthesis sizes (*p* < 0.05). Patients with 19 mm prostheses had significantly higher postoperative standardized mean gradient values than those with 21 mm prostheses.

Among patients who received the 21 mm prostheses, no significant differences were observed in postoperative LVEF, postoperative maximal gradient, postoperative mean gradient, postoperative septal thickness, or postoperative ventricular free wall thickness between those implanted with SJM HP and those implanted with SJM Regent prostheses (*p* > 0.05).

However, a significant difference was detected in postoperative standardized maximal gradient values between the two prosthesis models (*p* < 0.001). Patients who received SJM HP prostheses exhibited significantly higher postoperative standardized maximal gradient values compared to those who received SJM Regent prostheses.

Likewise, a significant difference was found in postoperative standardized mean gradient values between the two prosthesis models (*p* < 0.001). Patients with SJM HP prostheses had significantly higher postoperative standardized mean gradient values compared to those with SJM Regent prostheses.

According to Table 4, no significant differences were observed in intensive care unit (ICU) stay duration or total hospital stay duration among patients who underwent SJM HP implantation, SJM Regent implantation, or aortic root enlargement (*p* > 0.05).

However, a significant difference was found in patient–prosthesis mismatch (PPM) rates among the three groups (*p* < 0.001). The proportion of patients with moderate mismatch was significantly higher in the SJM HP group compared to both the SJM Regent and aortic-root-enlargement groups. Additionally, patients who underwent aortic root enlargement had a higher rate of moderate mismatch compared to those in the SJM Regent group.

Conversely, when considering the proportion of patients with no mismatch, this rate was highest in the SJM Regent group and lowest in the SJM HP group.

No significant differences were observed in mortality rates among patients in the SJM HP, SJM Regent, and aortic root enlargement groups (*p* > 0.05).

## 4. Discussion

The objective evaluation of prosthetic valve hemodynamics in patients undergoing mechanical aortic valve replacement is crucial for long-term prognosis and clinical management. Postoperative transvalvular gradient measurements are among the most widely used parameters for assessing prosthetic valve function and detecting complications such as patient–prosthesis mismatch. However, traditional gradient measurements are highly dependent on patient-specific factors such as prosthesis size, ejection fraction, and body surface area, leading to significant variability in the interpretation of hemodynamic outcomes [21,22,23,24]. This variability may result in misclassification of PPM severity and suboptimal prosthesis selection.

In the literature, PPM has been strongly associated with elevated postoperative transvalvular gradients and adverse long-term outcomes. Pibarot et al. demonstrated that higher postoperative gradients in patients with small prosthetic valves may contribute to left ventricular hypertrophy, increased cardiovascular morbidity, and reduced survival rates [25]. However, conventional gradient calculations fail to account for the patient’s individual hemodynamic profile, leading to inconsistent and potentially misleading clinical interpretations.

To overcome these limitations, our study introduced four standardized gradient calculation methods designed to adjust transvalvular gradient values based on critical hemodynamic factors. These methods aimed to minimize interpatient variability and provide a more objective assessment of prosthetic valve performance. Our findings suggest that standardized gradient calculations may improve assessment and reduce discrepancies in evaluating hemodynamic function.

Our study demonstrated that maximal and mean transvalvular gradient values significantly decreased postoperatively in all patient groups (*p* < 0.001), consistent with previous studies reporting a reduction in transvalvular gradients following AVR [26]. However, when traditional gradient measurements were used, substantial variation was observed based on prosthesis size, ejection fraction, effective orifice area, and body surface area, making direct comparisons between patient groups challenging. Conversely, standardized gradient calculations adjusted for these individual differences, allowing for more consistent and clinically meaningful comparisons [27].

Postoperatively, standardized maximum and mean gradients were significantly higher in the SJM HP group compared to both the SJM Regent and aortic-root-enlargement groups (*p* < 0.001 and *p* < 0.05, respectively), suggesting that prosthesis type significantly impacts postoperative hemodynamics and PPM risk [28]. Patients who underwent aortic root enlargement had significantly lower postoperative standardized gradients compared to the SJM HP group (*p* < 0.05), reinforcing its role in mitigating PPM-related complications [29]. These findings suggest that root enlargement may be a useful surgical strategy in patients at high risk for mismatch.

The proportion of female patients was higher in the aortic root enlargement group compared to the SJM HP and SJM Regent groups. This can be explained by the smaller anatomical aortic root diameter in female patients, necessitating root enlargement to accommodate the prosthetic valve. This emphasizes the importance of considering anatomical variations during prosthesis selection and surgical planning to optimize postoperative hemodynamics.

While conventional gradient measurements did not show a significant difference, standardized calculations revealed that patients with 19 mm prostheses exhibited significantly higher postoperative gradients compared to those with 21 mm prostheses (*p* < 0.05). This finding suggests that conventional measurements may underestimate the hemodynamic impact of prosthesis size, which could lead to suboptimal valve selection in clinical practice. Although aortic root enlargement should not be performed indiscriminately, the risk of increased postoperative gradients in smaller prostheses should be carefully considered when making surgical decisions [30].

PPM rates varied significantly among the groups (*p* < 0.001). Patients with SJM HP valves had a higher incidence of moderate PPM compared to those with SJM Regent or aortic root enlargement procedures, further reinforcing the role of root enlargement as an effective approach for optimizing hemodynamic performance in at-risk patients. Given that PPM has been associated with higher long-term mortality and increased risk of heart failure, early identification of at-risk patients is essential. The increased incidence of moderate mismatch in the SJM HP group highlights the necessity of careful prosthesis selection—particularly in patients with a small annulus—to avoid unnecessary hemodynamic burden postoperatively [31].

These four standardized gradient calculation methods address the limitations of conventional gradient measurements by minimizing the impact of patient-specific variables, thus allowing for a more accurate evaluation of prosthetic valve performance. For example, when two patients with similar ejection fractions, body surface areas, and postoperative gradient values receive prosthetic valves of the same size—one with an SJM Regent and the other with an SJM HP—standardized calculations reveal that the postoperative gradient in the Regent valve is lower than in the HP valve. This demonstrates how traditional gradient assessments may overlook clinically significant differences between prosthetic valve types. Our findings suggest that standardized calculations provide a more reliable assessment of postoperative hemodynamics, particularly in patients at risk of PPM. The ability to correct for interpatient variability enhances clinical decision-making and may help optimize prosthesis selection and surgical strategies to improve long-term outcomes.

Although this study provides important insights, certain limitations should be acknowledged. First, the retrospective nature of our study introduces potential selection bias and limits control over confounding variables. Future prospective randomized controlled trials are necessary to validate these findings. Second, this is a single-center study conducted at Ankara Bilkent City Hospital, and the results may not be generalizable to other populations. Large-scale multi-center studies are required to confirm the validity and reliability of standardized gradient calculation methods. Third, the sample size is relatively limited (115 patients divided into three groups), restricting the generalizability of our findings, particularly when making subgroup comparisons (e.g., 19 mm vs. 21 mm prostheses). The impact of standardized gradient calculations on clinical outcomes should be further evaluated in larger cohorts. Fourth, our study lacks long-term follow-up data. While we analyzed preoperative and early postoperative gradient changes, the long-term effects on ventricular function, patient symptoms, and clinical prognosis remain unknown. Future studies should examine the relationship between standardized gradient calculations and long-term follow-up data to determine their predictive value in clinical outcomes. Fifth, gradient measurements were performed using transthoracic echocardiography. More advanced imaging techniques, such as transesophageal echocardiography or cardiac magnetic resonance, were not included. Comparative studies using different imaging modalities could provide a more comprehensive evaluation of patient–prosthesis mismatch. Sixth, observer-related variability in echocardiographic measurements should be considered. Although intra-observer and inter-observer reliability analyses showed excellent agreement (ICC: 0.89–0.91), small variations in transvalvular gradient measurements could still influence individual patient assessments. Future studies should investigate whether additional automated or AI-assisted echocardiographic analysis techniques could further enhance measurement consistency. Seventh, the four standardized gradient calculation methods introduced in this study have not yet been externally validated in independent cohorts. While these methods were designed to minimize interpatient variability, their generalizability across different patient populations, prosthetic valve types, and clinical settings remains unknown. Further studies involving multi-center validation and prospective trials are needed to confirm their clinical utility.

Finally, our study focused exclusively on mechanical prosthetic valves, and bioprosthetic valves were not evaluated. Whether these standardized calculations apply to bioprosthetic valves remains unknown. Future research should explore their applicability to bioprosthetic valve populations to enhance clinical decision-making.

## 5. Conclusions

This study demonstrates that standardized gradient calculations provide a more consistent and objective assessment of prosthetic valve function in mechanical aortic valve replacement (AVR) by minimizing interpatient variability. These calculations may improve the detection of patient–prosthesis mismatch (PPM); however, further validation in larger, multi-center studies is required before widespread clinical implementation. One of the key findings of this study is the significant difference in standardized gradient values between 19 mm and 21 mm prostheses, emphasizing the impact of prosthesis size on postoperative hemodynamics. Unlike conventional gradient measurements, standardized calculations incorporate body surface area, ejection fraction, and effective orifice area, enabling more accurate comparisons and reducing misinterpretations due to individual variability. Additionally, our results confirm that aortic root enlargement (ARE) significantly lowers standardized postoperative gradients, supporting its role in mitigating PPM risk in selected patients. Among prosthetic valve types, SJM Regent valves demonstrated better hemodynamic performance than SJM HP valves, particularly in patients who underwent aortic root enlargement. The four standardized gradient calculation methods introduced in this study offer a clinically relevant approach to prosthetic valve evaluation, potentially enhancing surgical decision-making and prosthesis selection. However, their clinical utility must be confirmed through further prospective multi-center studies with larger patient cohorts and long-term follow-up. In conclusion, while standardized gradient calculations show promise in improving prosthetic valve assessment, further validation and refinement are required before they can be widely adopted into clinical practice.

## Figures and Tables

**Table 1:** Comparison of the characteristics of patients undergoing SJM HP, SJM Regent, and root enlargement.

	SJM HP (*n* = 31)		SJM Regent (*n* = 54)		Root Enlargement (*n* = 30)		
	Mean ± SD Median (Min–Max)		Mean ± SD Median (Min–Max)		Mean ± SD Median (Min–Max)		*p*
Age	57.16 ± 13.34 61 (23–75)		55.66 ± 11.41 59 (18–74)		52.53 ± 17.32 59 (18–76)		0.581 ^b^
Height	163.29 ± 8.41		164.11 ± 9.47		161.53 ± 10.09		0.484 ^d^
Weight	72.52 ± 10.89		73.33 ± 11.00		73.33 ± 13.61		0.946 ^d^
BMI (kg/m^2^)	27.29 ± 4.21		27.37 ± 4.55		28.27 ± 5.72		0.421 ^d^
Body surface area m^2^	1.78 ± 0.15		1.80 ± 0.15		1.80 ± 0.15		0.160 ^d^
Preop aortic root	2.12 ± 0.12 2.1 (1.8–2.4)		2.13 ± 0.17 2.1 (1.8–2.6)		2.08 ± 0.11 2.1 (1.9–2.3)		0.305 ^b^
Preop AVA	0.76 ± 0.12 0.8 (0.6–1.0)		0.75 ± 0.16 0.8 (0.5–1.2)		0.73 ± 0.15 0.7 (0.5–1.1)		0.707 ^b^
Prosthesis effective orifice area (cm^2^)	1.36 ± 0.12 1.4 (1.0–1.4)		1.90 ± 0.17 2.0 (1.6–2.0)		1.74 ± 0.28 1.8 (1.0–2.0)		<0.001 ^b^
	*n*	%	*n*	%	*n*	%	
Gender							
Female	9	29.0	23	42.6	20	66.7	0.011 ^c^
Male	22	71.0	31	57.4	10	33.3	
Morbidity							
No	31	100	51	94.4	28	93.3	0.488 ^c^
Yes	0	0	3	5.6	2	6.7	
HT							
No	7	22.6	22	40.7	10	33.3	0.234 ^c^
Yes	24	77.4	32	59.3	20	66.7	
DM							
No	24	77.4	38	70.4	23	76.7	0.716 ^c^
Yes	7	22.6	16	29.6	7	23.3	
Chronic kidney disease							
No	30	96.8	51	94.4	30	100	0.807 ^c^
Yes	1	3.2	3	5.6	0	0	
COPD							
No	26	83.9	45	83.3	28	93.3	0.446 ^c^
Yes	5	16.1	9	16.7	2	6.7	
Valve size							
19	3	9.7	13	24.1	9	30.0	0.134 ^c^
21	28	90.3	41	75.9	21	70.0	

BMI: body mass index; Preop: preoperative; AVA: aortic valve area; HT: hypertension; DM: diabetes mellitus; COPD: chronic obstructive pulmonary disease. Statistical tests: b: Kruskal–Wallis variance analysis; d: one-way Analysis of Variance (ANOVA); c: Chi-Square test/Fisher’s exact test.

**Table 2:** Comparison of preoperative measurements and postoperative measurements (within-group) and postoperative measurements (between groups) of patients who underwent SJM HP, SJM Regent, and root enlargement.

		Preoperative	Postoperative	
	Group	Mean ± SD Median (Min–Max)	Mean ± SD Median (Min–Max)	*p*
LVEF	SJM HP	56.65 ± 8.51 60 (32–70)	58.45 ± 6.15 60 (38–75)	0.237 ^f^
	SJM Regent	57.87 ± 7.61 60 (30–70)	56.54 ± 6.99 60 (25–65)	0.032 ^f^
	Root enlargement	59.00 ± 7.77 60 (35–78)	59.40 ± 6.21 60 (47–78)	0.837 ^f^
*p*			0.220 ^b^	
Δ LVEF	SJM HP	1.80 ± 6.76; 0 (−12)–(20.0)		N/A
	SJM Regent	−1.33 ± 5.24; 0 (−15)–(14)		N/A
	Root enlargement	0.40 ± 6.30; 0 (−15)–(13)		N/A
*p*		0.075 ^b^		
Maximal gradient (mm Hg)	SJM HP	76.97 ± 17.01 70 (60–133)	22.35 ± 5.81 21 (14–40)	<0.001 ^f^
	SJM Regent	83.63 ± 22.54 75 (52–165)	21.50 ± 6.12 20 (12–34)	<0.001 ^f^
	Root enlargement	83.90 ± 22.13 78 (52–144)	23.40 ± 6.39 23 (13–43)	<0.001 ^f^
*p*			0.365 ^b^	
Δ Maximal gradient	SJM HP	−54.61 ± 16.79; −52 (−98)–(−30)		N/A
	SJM Regent	−62.12 ± 23.14; 59 (−135)–(−22)		N/A
	Root enlargement	−60.52 ± 22.19; −57 (−116)–(−25)		N/A
		0.335 ^b^		
Mean gradient (mm Hg)	SJM HP	47.97 ± 10.76 44 (31–78)	12.26 ± 3.34 12 (7–20)	<0.001 ^f^
	SJM Regent	52.22 ± 16.16 47 (30–98)	11.59 ± 3.66 11 (5–20)	<0.001 ^f^
	Root enlargement	50.83 ± 15.24 45 (30–95)	12.50 ± 3.35 12 (7–23)	<0.001 ^f^
*p*			0.384 ^b^	
Δ Mean gradient	SJM HP	−35.70 ± 10.93; −35 (−64)–(−18)		N/A
	SJM Regent	−40.62 ± 16.69; −38 (−84)–(−15)		N/A
	Root enlargement	−38.33 ± 15.08; −33 (−82)–(−18)		N/A
		0.525 ^b^		
Septum thickness (cm)	SJM HP	1.30 ± 0.16 1.3 (1.0–1.6)	1.65 ± 0.11 1.2 (1.0–1.4)	<0.001 ^f^
	SJM Regent	1.33 ± 0.22 1.3 (0.7–2.0)	1.20 ± 0.16 1.2 (0.8–1.5)	<0.001 ^f^
	Root enlargement	1.28 ± 0.27 1.2 (0.8–2.2)	1.17 ± 0.22 1.1 (0.8–1.9)	<0.001 ^f^
*p*			0.154 ^b^	
Δ Septum thickness	SJM HP	−0.13 ± 0.13; −0.1 (−0.5)–(0)		N/A
	SJM Regent	−0.12 ± 0.16; −0.1 (−0.7)–(0.3)		N/A
	Root enlargement	−0.10 ± 0.15; −0.1 (−0.6)–(0.1)		N/A
		0.397 ^b^		
Free wall thickness (cm)	SJM HP	1.22 ± 0.15 1.2 (1.0–1.5)	1.12 ± 0.12 1.1 (0.9–1.4)	<0.001 ^f^
	SJM Regent	1.22 ± 0.18 1.2 (0.7–1.6)	1.13 ± 0.13 1.1 (0.8–1.5)	<0.001 ^f^
	Root enlargement	1.14 ± 0.18 1.1 (0.9–1.8)	1.09 ± 0.17 1.0 (0.9–1.8)	0.027 ^f^
*p*			0.139 ^b^	
Δ Free wall thickness	SJM HP	−0.10 ± 0.12; −0.1 (−0.3)–(0.2)		N/A
	SJM Regent	−0.08 ± 0.14; −0.1 (−0.4)–(0.2)		N/A
	Root enlargement	−0.04 ± 0.15; 0 (−0.4)–(0.5)		N/A
		0.290 ^b^		
Standardized maximum gradient	SJM HP	22.49 ± 7.94 21.52 (12.38–46.70)	3.32 ± 0.83 3.28 (1.84–5.46)	<0.001 ^f^
	SJM Regent	24.90 ± 12.02 21.79 (11.45–58.33)	2.35 ± 0.87 2.22 (1.18–6.49)	<0.001 ^f^
	Root enlargement	24.41 ± 10.66 20.94 (1.69–50.52)	2.70 ± 1.11 2.61 (1.25–7.10)	<0.001 ^f^
*p*			<0.001 ^b^	
Δ Standardized maximum gradient	SJM HP	−19.16 ± 7.98; 17.30 (−42.41)–(−8.76)		N/A
	SJM Regent	−22.54 ± 11.98; −19.58 (−55.44)–(−8.16)		N/A
	Root enlargement	−21.70 ± 10.63; −18.35 (−47.30)–(−9.5)		N/A
*p*		0.695 ^b^		
Standardized mean gradient	SJM HP	14.08 ± 5.48 13.73 (7.68–34.41)	1.83 ± 0.55 1.82 (0.92–3.15)	<0.001 ^f^
	SJM Regent	15.58 ± 8.01 12.59 (6.70–37.07)	1.26 ± 0.47 1.19 (0.47–2.95)	<0.001 ^f^
	Root enlargement	14.86 ± 6.97 11.75 (6.15–31.58)	1.44 ± 0.58 1.32 (0.72–3.80)	<0.001 ^f^
*p*			<0.001 ^b^	
Δ Standardized mean gradient	SJM HP	−12.24 ± 5.41; −11.03 (−31.65)–(−5.66)		N/A
	SJM Regent	−14.31 ± 7.98; −11.46 (−35.8)–(−5.56)		N/A
	Root enlargement	−13.42 ± 6.95; −10.54 (−29.62)–(−4.83)		N/A
		0.777 ^b^		

LVEF: left ventricular ejection fraction; Δ: change values (postoperative–preoperative). Statistical tests: f: Wilcoxon’s test; b: Kruskal–Wallis variance analysis.

**Table 3:** Comparison of patients with prosthesis sizes 19 mm and 21 mm and postoperative measurements in patients with SJM Regent.

	SJM Regent 19 mm (*n* = 13)	SJM Regent 21 mm (*n* = 41)	
	Mean ± SD Median (Min–Max)	Mean ± SD Median (Min–Max)	*p*
Postoperative LVEF	55.38 ± 6.60 60 (40–60)	56.90 ± 7.15 60 (25–65)	0.407 ^h^
Postoperative maximal gradient	22.00 ± 6.96 21 (12–34)	21.34 ± 5.91 20 (14–34)	0.670 ^h^
Postoperative mean gradient	12.15 ± 4.07 11 (7–20)	11.41 ± 3.55 11 (5–20)	0.626 ^h^
Postoperative septum thickness	1.16 ± 0.12 1.1 (1.0–1.4)	1.22 ± 0.16 1.2 (0.8–1.5)	0.104 ^h^
Postoperative free wall thickness	1.10 ± 0.10 1.1 (1.0–1.3)	1.14 ± 0.14 1.1 (0.8–1.5)	0.396 ^h^
Postoperative standardized maximum gradient	2.89 ± 1.23 2.77 (1.53–6.49)	2.18 ± 0.65 2.10 (1.18–3.43)	0.025 ^h^
Postoperative standardized mean gradient	1.58 ± 0.59 1.39 (0.89–2.95)	1.16 ± 0.38 1.10 (0.47–1.88)	0.021 ^h^
	**SJM HP** **21 mm (*n* = 28)**	**SJM Regent** **21 mm (*n* = 41)**	
Postoperative LVEF	58.36 ± 6.47 60 (38–75)	56.90 ± 7.15 60 (25–65)	0.341 ^h^
Postoperative maximal gradient	22.36 ± 6.06 20 (14–40)	21.34 ± 5.91 20 (14–34)	0.454 ^h^
Postoperative mean gradient	12.21 ± 3.45 12 (7–20)	11.41 ± 3.55 11 (5–20)	0.329 ^h^
Postoperative septum thickness	1.15 ± 0.12 1.2 (1.0–1.4)	1.22 ± 0.16 1.2 (0.8–1.5)	0.066 ^h^
Postoperative free wall thickness	1.13 ± 0.12 1.1 (1.0–1.4)	1.14 ± 0.14 1.1 (0.8–1.5)	0.745 ^h^
Postoperative standardized maximum gradient	3.18 ± 0.71 3.19 (1.84–4.88)	2.18 ± 0.65 2.10 (1.18–3.43)	<0.001 ^h^
Postoperative standardized mean gradient	2.18 ± 0.65 2.10 (1.18–3.43)	1.16 ± 0.38 1.10 (0.47–1.88)	<0.001 ^h^

LVEF: left ventricular ejection fraction. Statistical tests: h: Mann–Whitney U test.

**Table 4:** Comparison of ICU length of stay, length of hospital stay, mortality, and non-compliance rates of patients who underwent SJM HP, SJM Regent, and root enlargement.

	SJM HP (*n* = 31)		SJM Regent (*n* = 54)		Root Enlargement (*n* = 30)		
	Mean ± SD Median (Min–Max)		Mean ± SD Median (Min–Max)		Mean ± SD Median (Min–Max)		*p*
ICU length of stay	1.19 ± 0.60 1 (1–3)		1.43 ± 1.62 1 (1–12)		1.43 ± 0.81 1 (1–4)		0.219 ^b^
Length of hospital stay	6.19 ± 1.85 6 (4–13)		6.22 ± 2.40 6 (4–19)		6.90 ± 3.82 6 (0–20)		0.610 ^b^
	***n***	**%**	***n***	**%**	***n***	**%**	
Incompatibility							
Unimportant	10	32.3	53	98.1	24	80.0	<0.001 ^c^
Moderate	20	64.5	1	1.9	6	20.0	
Severe	1	3.2	0	0	0	0	
Mortality							
None (alive)	31	100	53	98.1	29	96.7	0.745 ^c^
Var (ex)	0	0	1	1.9	1	3.3	

ICU: intensive care unit. Statistical tests: b: Kruskal–Wallis variance analysis; c: Chi-Square test/Fisher’s exact test.

## Data Availability

The raw data supporting the conclusions of this article will be made available by the authors upon request.

## Abbreviations

AS	Aortic stenosis
AVR	Aortic valve replacement
PPM	Patient–prosthesis mismatch
TTE	Transthoracic echocardiography
LVEF	Left ventricular ejection fraction
BSA	Body surface area
EOA	Effective orifice area
AVA	Aortic valve area
ARE	Aortic root enlargement
MaxG	Maximum transvalvular gradient
MeanG	Mean transvalvular gradient
CW Doppler	Continuous-wave Doppler
Std-MaxG Preop	Preoperative standardized maximum gradient
Std-MeanG Preop	Preoperative standardized mean gradient
Std-MaxG Postop	Postoperative standardized maximum gradient
Std-MeanG Postop	Postoperative standardized mean gradient
D(Ao)	Aortic root diameter
D(Pr)	Prosthetic valve diameter
SJM HP	St. Jude Medical High-Performance Prosthetic Valve
SJM Regent	St. Jude Medical Regent Prosthetic Valve
ICU	Intensive care unit
NYHA	New York Heart Association
